# New models of Parkinson’s like neuroinflammation in human microglia clone 3: Activation profiles induced by INF-γ plus high glucose and mitochondrial inhibitors

**DOI:** 10.3389/fncel.2022.1038721

**Published:** 2022-11-29

**Authors:** Francesca De Chirico, Eleonora Poeta, Giorgia Babini, Iliana Piccolino, Barbara Monti, Francesca Massenzio

**Affiliations:** Department of Pharmacy and Biotechnology, University of Bologna, Bologna, Italy

**Keywords:** human microglia, HMC3, neuroinflammation, Parkinson’s disease, IFN-γ, glucose, 6-OHDA, MPTP

## Abstract

Microglia activation and neuroinflammation have been extensively studied in murine models of neurodegenerative diseases; however, to overcome the genetic differences between species, a human cell model of microglia able to recapitulate the activation profiles described in patients is needed. Here we developed human models of Parkinson’s like neuroinflammation by using the human microglia clone 3 (HMC3) cells, whose activation profile in response to classic inflammatory stimuli has been controversial and reported only at mRNA levels so far. In fact, we showed the increased expression of the pro-inflammatory markers iNOS, Caspase 1, IL-1β, in response to IFN-γ plus high glucose, a non-specific disease stimulus that emphasized the dynamic polarization and heterogenicity of the microglial population. More specifically, we demonstrated the polarization of HMC3 cells through the upregulation of iNOS expression and nitrite production in response to the Parkinson’s like stimuli, 6-hydroxidopamine (6-OHDA) and 1-methyl-4-phenyl-1,2,3,6-tetrahydropyridine (MPTP), the latter depending on the NF-κB pathway. Furthermore, we identified inflammatory mediators that promote the pro-inflammatory activation of human microglia as function of different pathways that can simulate the phenotypic transition according to the stage of the pathology. In conclusion, we established and characterized different systems of HMC3 cells activation as *in vitro* models of Parkinson’s like neuroinflammation.

## Introduction

Microglia are the only myeloid, resident immune cells in the brain, ensuring the environmental homeostasis and the regulation of synaptic functions (Sousa et al., 2017; Augusto-Oliveira et al., 2019). The first response of microglia to neurodegenerative diseases’ onset is driven by the anti-inflammatory phenotype (Xu et al., 2015) followed by the activation toward a pro-inflammatory one which results in the production of mediators that exacerbate the neuronal damage (Jurga et al., 2020).

Upon activation, microglia undergo phenotypic and morphological changes, and a metabolic switch from oxidative phosphorylation to aerobic glycolysis that reinforce the immune response through a “trained-immunity” phenotype (Cheng et al., 2014; Gimeno-Bayón et al., 2014; Orihuela et al., 2016). Indeed, high glucose enhances LPS-mediated activation in macrophages through the upregulation of TLR4, and the following increased expression of iNOS and NF-κB(p65), as well as IFN-γ-mediated activation through the JAK/STA1 pathway followed by the expression of metalloproteinases1, IL-1β and the nitric oxide production *via* the activation of Cx43 hemichannels in endothelial cell (Nareika et al., 2009; Sáez et al., 2020; Hung et al., 2022).

Microgliosis is a common feature of the neuroinflammatory process present in almost all neurodegenerative diseases, including Parkinson’s disease (PD) (Tanaka et al., 2013). Indeed, pro-inflammatory cytokines have been found in the serum from PD patients with increased concentration of IFN-γ (Liu et al., 2003; Mount et al., 2007; Scalzo et al., 2009). The discovery that mitochondrial respiratory chain inhibition led to Parkinson’s like symptoms, paved the way for the use of neurotoxins like 6-hydroxidopamine (6-OHDA) or 1-methyl-4-phenyl-1,2,3,6-tetrahydropyridine (MPTP) or Rotenone, to recapitulate the PD hallmarks (Giordano et al., 2012; Haas et al., 2016; Maegawa and Niwa, 2021). In fact, *in vitro* and *in vivo* studies showed a significant microgliosis that led to neurodegeneration (Wu et al., 2002; Zhang C. et al., 2021), prevented by immunomodulatory treatments (He et al., 2001; Yan et al., 2015; Zhang et al., 2017).

Although rodent models of microglia are widespread, gene changes of human inflammatory conditions do not necessarily correlate with human genomic changes (Seok et al., 2013; Galatro et al., 2017). It is the case of IBA1 and the transcription factor PU.1, similarly expressed in microglia across different species (Smith et al., 2013b), and the TLR4 whose expression is lower in human microglia compared to rodent cells (Carpentier et al., 2008; Parajuli et al., 2012), as well as the low to absent expression of CD14 or TNFα (Dello Russo et al., 2018; Jurga et al., 2020). Similarly, the pro-inflammatory marker iNOS, promptly activated in rodent microglia upon inflammatory stimuli (Sierra et al., 2014; Lively and Schlichter, 2018), do not share a similar accurate response in human cells (Janabi, 2002; Li et al., 2009; Hjorth et al., 2010; Landry et al., 2012; Jadhav et al., 2014), along with the proliferation rate of cultured microglia (Gibbons et al., 2007; Li et al., 2013; Smith et al., 2013a; Smith and Dragunow, 2014; Cavaliere et al., 2020).

To overcome the limitations of the murine models we enrolled the human microglia clone 3 (HMC3) cell line HMC3, established in the laboratory of Prof. Tardieu in 1995 and further characterized by upregulation of microglial markers upon IFN-γ treatment and less responsiveness to inflammatory stimuli compared to murine cells (Janabi et al., 1995; Dello Russo et al., 2018).

Here we treated HMC3 cells with IFN-γ plus high glucose, and Parkinson’s like stimuli to induce and characterize the phenotypic shift toward the pro-inflammatory one without affecting the phagocytic properties.

## Materials and methods

### Human microglia clone 3 cell cultures and treatments

Human microglia clone 3 cells were cultured in DMEM High Glucose Medium (4.5 g/l), 10% FBS-HI, 1% penicillin/streptomyces (all from Euroclone, Milano, Italy), in humified atmosphere of 5% CO_2_. HMC3 cells were treated with high glucose to the final concentration of 5 g/l w/or w/o IFN-γ (1 μg/ml), or increasing concentration of 6-OHDA (1 and 10 μM), or MPTP (0,01, 0,1 and 1 μM), in serum free medium for 24 h. Lysed cells and conditioned media were collected and stored for the following analysis.

### Western blot analysis

To analyze protein release, 500 μl of conditioned media (CM) were concentrated using Amicon YM-3 centrifuge filters (Millipore, Burlington, MA, United States) and resuspended in 4X Loading Buffer (0.1 M Tris HCl pH 6.8, 20%SDS, 0.4 μl/ml glycerol, 2 *g*/l bromophenol blue, 2 mM dithiothreitol; Sigma-Aldrich, St Louis, MO, United States). Cells were collected in lysis buffer (1%SDS, 50 mM Tris HCl pH7.4, 1 mM EDTA, 10 μl/ml protease inhibitors, 10 μl/ml phosphatase inhibitors) and protein content determined (Lowry et al., 1951). CM and cell samples (30 μg total protein) were loaded into 10% SDS-polyacrylamide gels (Bio-Rad, Hercules, CA, United States). Electrophoresis was followed by transfer onto nitrocellulose membranes (GE Healthcare, Milano, Italy), blocking in 5% non-fat dry milk (Bio-Rad, Hercules, CA, United States) in PBS-0.1% Tween-20 and incubation overnight at 4 °C with the following primary antibodies: iNOS, NF-κB(p65), and NF-κB, IL-1β (all 1:1,000 dilution, Cell Signaling Technology, Danvers, Massachusetts), TREM2 (1:1,000 dilution, Thermo-Fisher, Waltham, MA, United States), Arginase1, Caspase1, and GAPDH (1:1,000, the latter 1:20,000 dilution, Santa Cruz Biotechnology, Dallas, TX, United States). Membranes were then incubated with HFP-conjugated secondary antibodies in PBS-0.1% Tween-20 (1:5,000 dilution, Jackson ImmunoResearch, West Grove, PA, United States). Labeled proteins were visualized by using the Clarity™-Western ECL Substrate (Bio-Rad, Hercules, CA, United States) and detected using the ChemiDoc™ MP imaging system (Bio-Rad, Hercules, CA, United States). Densitometric analysis was performed by using Bio-rad Image Lab software (Version 6.1).

### Immunofluorescence staining

A total of 24 h after treatments with IFN-γ (1 μg/ml) plus 5 g/l glucose or 6-OHDA 10 μM, or MPTP 1 μM, cells were fixed with 4% paraformaldehyde in PB 0.1%, pH7.4 for TREM2 staining or cold methanol for IBA1, the latter followed by antigen retrieval in 10 mM Sodium Citate pH 6. Non-specific sites were blocked by incubation in PBS-0.1% TritonX-5% normal goat serum for TREM2 and in PBS-0.1% TritonX-5% BSA, 22.52 mg/ml glycine for IBA1 before the overnight incubation with primary antibodies, respectively 1:50 and 1:500 dilution in PBS-0.1% TritonX-2% goat serum. Then, fixed cells were incubated with fluorophore-conjugated secondary antibodies (Alexa Fluor Dyes, Abcam, Cambridge, United Kingdom). Nuclei were stained with Hoechst 33,258 and images acquired with a Nikon EZ-C1 confocal microscope with 100X oil immersion objective to quantify the mean fluorescence intensity of TREM2 and IBA1 and microglial morphology as function of cell size (area, perimeter, and diameter) and roundness by using the ROI management plugin and measurement setting of Fiji (ImageJ) software (all reagents from Sigma-Aldrich, St Louis, MO, United States).

### Nitrite detection assay

Nitrite in CM from HMC3 exposed to IFN-γ (1 μg/ml) plus 5 g/l glucose or 6-OHDA (0, 1, and 10 μM) or MPTP (0, 0.01, 0.1, and 1 μM) was measured by a colorimetric assay based on the Griess reaction (Vargas-Maya et al., 2021).

### Statistical analysis

Data were analyzed by using the GraphPad Prism8, San Diego, CA, United States software and expressed as a mean ± standard error of independent experiments. One-way ANOVA followed by Dunnett’s *post-hoc* was used to compare the means between control and treated cells.

## Results

### IFN-γ plus high glucose induces the activation of human microglia clone 3 cells

**FIGURE 1:**
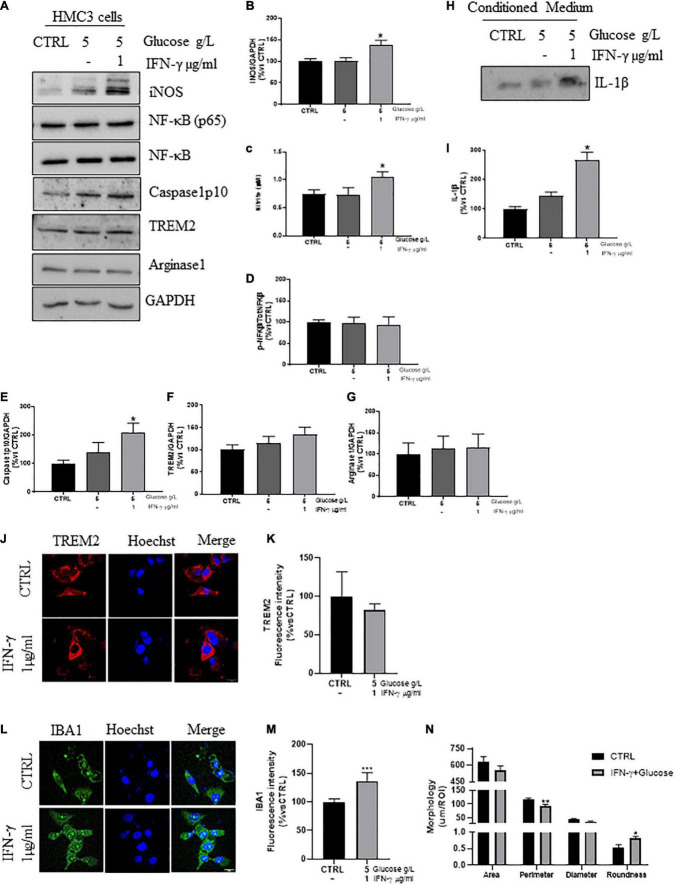
Phenotypic markers expression in human microglial clone 3 cell line (HMC3) treated with IFN-γ + glucose. Western blot analysis and relative densitometry of iNOS **(A,B)**, Caspase1 **(A,E)**, NF-κB(p65)/NF-κB **(A,D)**, TREM2 **(A,F)**, Arginase1 **(A,G)**, in lysed HMC3 cells treated with glucose (final concentration 5 g/l) or IFN-γ 1 μg/ml + glucose (final concentration 5 g/l) for 24 h in serum free medium vs. the reference protein GAPDH. IL-1β expression **(H,I)** and nitrite quantification **(C)** in conditioned medium of HMC3 cell treated with glucose (final concentration 5 g/l) or IFN-γ 1 μg/ml + glucose (final concentration 5 g/l) for 24 h. Results are the mean ± s.e. of independent experiments. *N* = 3 for Caspase1, NF-κB(p65)/NF-κB, IBA1, and IL-1β. *N* = 5 for iNOS, TREM2, and Arginase1. Representative immunofluorescence and relative quantification of fluorescence intensity of TREM2 **(J,K)**, and IBA1 **(L,M)** and morphology analysis of IBA1^+^ cells **(L,N)** in HMC3 cells treated with IFN-γ 1 μg/ml + glucose (final concentration 5 g/l) for 24 h. *N* = 3. Scale bar = 20 μM. One-way ANOVA, followed by Dunnett’s test *p < 0.05, **p < 0.01, and ***p < 0.001 vs. CTRL (glucose 4,5 g/l).

Since the classic activation stimulus LPS, commonly used in rodent cells, was not able to induce iNOS upregulation in HMC3 cells (Supplementary Figures 1A,B,C), slightly increased by IFN-γ (1 μg/ml) treatment (Supplementary Figures 1B,C), we stimulated the cells with IFN-γ plus high glucose. HMC3 were routinely cultured in DMEM High Glucose (4.5 g/l). We increased the amount of glucose to the final concentration of 5 g/l, which was not able to promote the increase of iNOS expression by itself (Supplementary Figures 1D,E), but significantly enhanced it in cells primed by IFN-γ (1 μg/ml, Figures 1A,B), accompanied by increased expression of Caspase1 (Figures 1A,E) and a comparable increase in nitrite (Figure 1C) and IL-1β (Figures 1H,J) release (Brough and Rothwell, 2007). Although NF-kB-dependent transcription of pro-inflammatory genes in macrophages has been reported following increased glycolytic rate (Shen et al., 2017), we did not observe any increase in the phosphorylation of NF-kB(p65) in our cells (Figures 1A,D). The heterogenicity of HMC3 cells characterized by globular and elongated, ramified morphology (Supplementary Figure 2A), was also found in the expression of phenotypic markers. Indeed, we detected a slight increasing trend in the expression of TREM2 in HMC3 cells treated with IFN-γ plus high glucose (Figures 1A,F), even though no changes in the quantification of fluorescence intensity were found (Figures 1J,K), as well as no differences in the expression of the M2a marker Arginase1 between control and treated cells were observed (Figures 1A,G).

Lastly, the increased fluorescence intensity of IBA1 in IFN-γ plus glucose-cells (Figures 1L,M), together with the reduced cell size accompanied by increased roundness, may indicated a reduced branching and hence ameboid morphology (Figures 1L,N).

### Parkinson’s like stimuli induce the activation of human microglia clone 3 cells

Given the key role of chronic inflammation in PD (Tanaka et al., 2013; Liu et al., 2022), we enrolled neurotoxin stimuli known to induce a Parkinson’s like phenotype to evaluate their effects on microglia activation and morphology (Dehmer et al., 2004; Gerhard et al., 2006; Silva et al., 2016; Zhang et al., 2017; Zhang D. et al., 2021).

As reported in Supplementary Figures 3, 4, neither treatment with increasing concentration of Rotenone (0, 0.01, and 0.1 μM), nor the priming effect of IFN-γ or glucose on rotenone, induced any modulation of microglial phenotype based on the markers observed. Thus, we investigated the contribution of 6-OHDA or MPTP in the modulation of HMC3 phenotype.

### 6-hydroxidopamine induces pro-inflammatory activation of microglia

**FIGURE 2:**
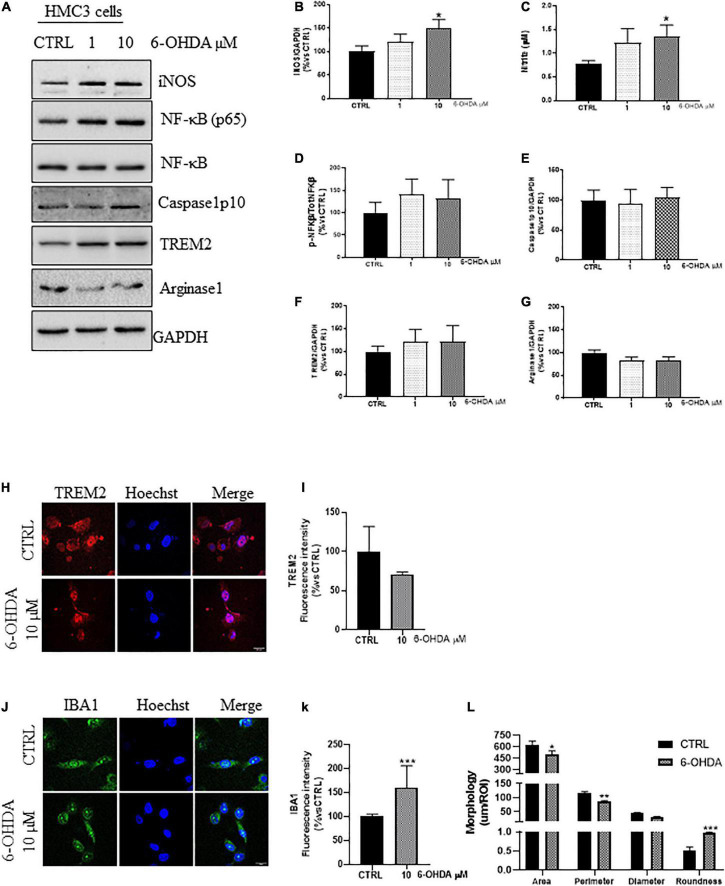
Phenotypic markers expression in human microglial clone 3 cell line (HMC3) treated with increasing concentration of 6-OHDA. Western blot analysis and relative densitometry of iNOS **(A,B)**, NF-κB(p65)/NF-κB **(A,D)**, Caspase1p10 **(A,E)**, TREM2 **(A,F)**, and Arginase1 **(A,G)**, in lysed cells vs. the reference protein GAPDH and nitrite quantification in conditioned medium **(C)** of HMC3 cell treated with increasing concentration of 6-OHDA (0, 1, and 10 μM) for 24 h. Results are the mean ± s.e. of independent experiments. *N* = 3 for Caspase1, NF-κB(p65)/NF-κB, and IBA1. *N* = 4 for TREM2, *N* = 5 for iNOS and Arginase1. Representative immunofluorescence and relative quantification of fluorescence intensity of TREM2 **(H,I)**, and IBA1 **(J,K)** and morphology analysis of IBA1^+^ cells **(J,L)** in HMC3 cells treated with 6-OHDA 10 μM for 24 h. *N* = 3. Scale bar = 20 μM. One-way ANOVA, followed by Dunnett’s test *p < 0,05, **p < 0.01, and ***p < 0.001 vs. CTRL.

Administration of 6-OHDA has been proved to increase the expression of pro-inflammatory markers both *in vitro* and *in vivo* (He et al., 2001; Yan et al., 2015; Zhang et al., 2017). We treated the cells with increasing concentration of 6-OHDA (0, 1, and 10 μM) for 24 h in serum free medium. 10 μM of 6-OHDA significantly increased the expression of iNOS (Figures 2A,B), and the nitrite release (Figure 2C), compared to untreated cells. Even though it has been reported the nuclear translocation of NF-κB(p65) in 6-OHDA-mice (Gasparotto et al., 2017) and its pro-apoptotic effect *in vitro* (Tarabin and Schwaninger, 2004), we detected only a slight, non-significant, increase in the *ratio* NF-kB(p65)/NF-κB (Figures 2A,D) in 6-OHDA-treated cells, with no upregulation of Caspase1 (Figures 2A,E), described to trigger the activation of the p38MAPK/NF-κB pathway and the inflammation-related pyroptosis in 6-OHDA rats and LPS-activated BV2 cells (Staal et al., 2011; Thawkar and Kaur, 2019; Cai et al., 2022). Concurrently, we showed no changes in the expression of TREM2 (Figures 2A,F,H,I) and Arginase1 (Figures 2A,G), supporting an early phenotypic shift toward the pro-inflammatory phenotype, also confirmed by the increased fluorescence intensity of IBA1 (Figures 2J,K), the increased roundness and the reduced size of IBA1^+^cells (Figures 2J,L) in 6-OHDA cells compared to control ones.

### 1-methyl-4-phenyl-1,2,3,6-tetrahydropyridine induces pro-inflammatory activation of microglia *via* the *NF-*κ*B pathway*

**FIGURE 3:**
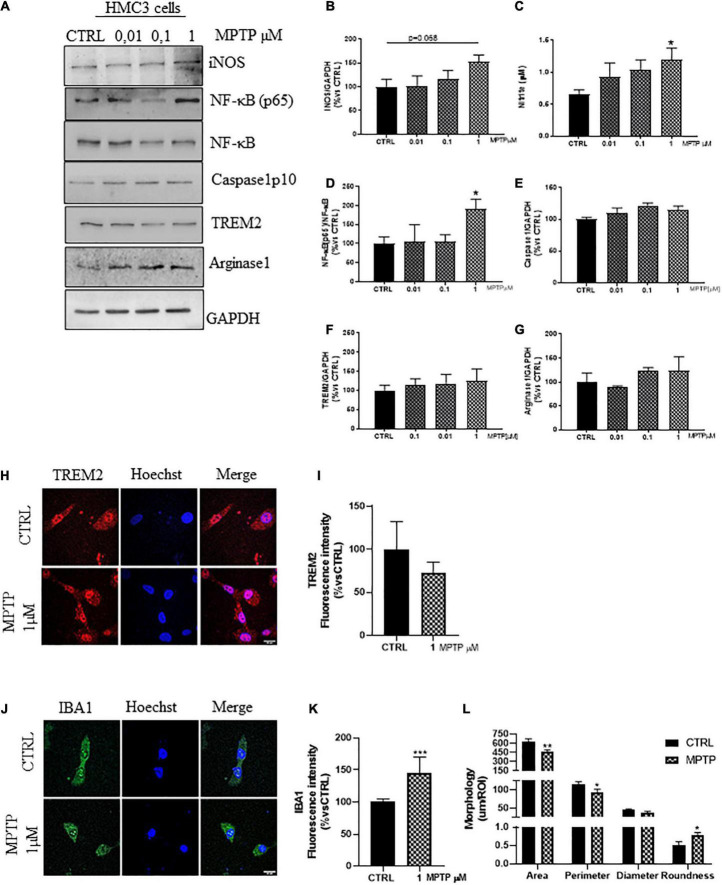
Phenotypic markers expression in human microglial clone 3 cell line (HMC3) treated with increasing concentration of MPTP. Western blot analysis and relative densitometry of iNOS **(A,B)**, NF-κB(p65)/NF-κB **(A,D)**, (Prati et al., 2015) Caspase1p10 **(A,E)**, TREM2 **(A,F)**, Arginase1 **(A,G)** in lysed cells vs. the reference protein GAPDH and nitrite quantification in conditioned medium **(C)** of HMC3 cell treated with increasing concentration of MPTP (0, 0,01, 0,1, and 1 μM) for 24 h. Results are the mean ± s.e. of three independent experiments. Representative immunofluorescence and relative quantification of fluorescence intensity of TREM2 **(H,I)**, and IBA1 **(J,K)** and morphology analysis of IBA1 cells **(J,L)** in HMC3 cells treated with MPTP 1 μM for 24 h. *N* = 3. Scale bar = 20 μM. One-way ANOVA, followed by Dunnett’s test *p < 0.05, **p < 0.01, and ***p < 0.001 vs. CTRL.

In rodents, MPTP produces neuropathological effects that mimic the loss of dopaminergic neurons and microglial activation observed in patients (Dehmer et al., 2000; Przedborski et al., 2000; Wu et al., 2002; Lee et al., 2019). Based on this, we treated the HMC3 cells with increasing concentrations of MPTP (0, 0.01, 0.1, and 1 μM) for 24 h in serum free medium (Supplementary Figure 5B). As demonstrated in Figures 3A,B, the MPTP treatment promoted a dose dependent increase of iNOS expression and nitrite release (Figure 3C), together with the significant increase in the *ratio* NF-kB(p65)/NF-kB (Figures 3A,D), resembling the NF-κB translocation observed in rat astrocytoma cell line (Niranjan et al., 2010). However, we observed no Caspase1 upregulation (Figures 3A,E), unlike the MPTP-mice (Qiao et al., 2017). The upcoming inflammation, could be partially counteracted by the unchanged expression (Figures 3A,F) and distribution (Figures 3H,I) of TREM2 and Arginase1 (Figures 3A,G; Ren et al., 2018; Guo et al., 2019) as confirmed by the increased fluorescence intensity of IBA1 (Figures 3J,K), and the morphological analysis of IBA1^+^cell (Figures 3J,L) upon MPTP treatment (1 μM), in which the reduced size and the increased roundness are attributable to an ameboid morphology.

## Discussion

Upon inflammatory stimuli, microglia undergo activation and release of pro-inflammatory mediators responsible for triggering chronic neuroinflammation widely described in neurodegenerative diseases (Zhang et al., 2017; Massenzio et al., 2018; Puntambekar et al., 2022). Therefore, exploiting the modulation of neuroinflammation for therapeutic purposes could represent a potential therapeutic route for neurodegenerative pathologies.

The HMC3 cells have been characterized by low to absent expression of CD14 and CD11b, similarly to human iPSC-derived microglia, positive for both macrophage markers IBA1 and Cx3cr1, and microglial ones TMEM119, P2RY12, and TREM2 (Bennett et al., 2016; Mildner et al., 2017; Pandya et al., 2017; Rai et al., 2020), as also confirmed by transcriptomic analysis (Galatro et al., 2017). Additionally, single cell RNA analysis identified the transcriptional profile of disease associated microglia (DAM), characterized by the expression of both immune cells and microglial markers. Markers of DAM, primarily found in Alzheimer’s disease (AD)-mouse models, were further identified in human AD tissues leading to speculate about the DAM phenotype as a common signature of microglial response to neurodegenerative pathologies (Butovsky and Weiner, 2018; Deczkowska et al., 2018). Indeed, a comparison analysis of gene expression profile of human and mouse iPSC-microglia in response to inflammatory stimuli, demonstrated a higher-than-expected overlap with genes that change their expression in microglia from AD patients and an unusually high number of protein interactions with the products of genes within genome-wide association study (GWAS) loci. However, the LPS-dependent response in mouse microglia required the priming effect of IFN-γ in human iPSC-microglia, corroborating a shared transcriptional response triggered by different inflammatory stimuli (Monzón-Sandoval et al., 2022). Similarly, LPS in combination with IFN-γ or ATP was reported by Dello Russo et al. (2018) to activate HMC3 cells.

We unveiled the activation profile of HMC3 cells in response to IFN-γ plus high glucose, since: (i) The canonical LPS treatment showed no effect on iNOS expression in our model, unlike IFN-γ treatment, which slightly increased it, supporting the priming role of IFN-γ in the process of microglia activation (Dello Russo et al., 2018; Ta et al., 2019; Wang et al., 2021), (ii) innate immune cells can adopt a “trained-immunity” phenotype, in which immune responses are reinforced through a boost in glycolysis (Cheng et al., 2014; Orihuela et al., 2016).

The ineffectiveness of LPS was not surprising, since it has been demonstrated that the expression of TLR4, is lower in human microglia compared to rodent cells (Saha and Pahan, 2006; Carpentier et al., 2008; Parajuli et al., 2012; Jurga et al., 2020).

The low to absent expression of CD14 and CD11b in HMC3 cells has been overcome by treatment with IFN-γ (Dello Russo et al., 2018; Wang et al., 2021). Even though the qRT-PCR has a higher sensitivity than the western blot we used, it does not take into account the differences between the prediction of the transcript and the subsequent protein expression (Janabi, 2002; Landry et al., 2012; Payne, 2015; Dello Russo et al., 2018; Chen et al., 2019; Buccitelli and Selbach, 2020; Chiu et al., 2020, 2021; Wang et al., 2021). Also, the low expression of CD14, might take advantage of to the priming role of IFN-γ. In fact, LPS alone can stimulate the CD14-inflammatory pathway in murine glial cells but not in human ones corroborating the need of priming human microglia (Suzuki et al., 2003; Saha and Pahan, 2006). Additionally, even though CD14 controls the LPS/TLR4 pathway, it was found to activate the MAPK-ERK1/ERK2/JNK pathway, resulting in an increased phagocytic activity to exert an initial beneficial role in deferring disease progression (Fu et al., 2014). Thus, we cannot exclude an early upregulation of CD14 resulting from a priming effect of IFN-γ that needs an additional incentive to move toward a pro-inflammatory condition. Additionally, CD11b was reported in both microglia and macrophage, whose phenotypic and functional distinction in human brain was due to the CD45 expression; thus justifying the slight upregulation of CD11b in response to IFN-γ in HMC3 cells, in conjunction with unchanged expression of TMEM 119 (Galatro et al., 2017; Rangaraju et al., 2018; Wang et al., 2021; Ruan and Elyaman, 2022). Anyway, the upregulation of CD14 and MHCII in HMC3 cells primed with IFN-γ, accompained by the upregulation of CD86 and CD68 and no changes in the expression of M2a markers, might be attributable to the intermediate M2b phenotype, also considering that none of the unquestionable pro-inflammatory markers have been observed (iNOS, IL-1β/Caspase1) (Zhou et al., 2017; Chiu et al., 2020; Jurga et al., 2020; Wang et al., 2021), endorsing the need of human cells to be primed with IFN-γ.

We demonstrated the pro-inflammatory activation of HMC3 cells, as function of iNOS expression and nitrite release, upon treatment with IFN-γ plus glucose compared to control condition and glucose by itself, as well as the increased secretion of IL-1β driven by the Caspase1 activity, as already demonstrated in IFN-γ-treated Th1 cells and primary microglia (Watanabe et al., 2016; Chen et al., 2018; Rui et al., 2020). High glucose enhanced the responsiveness to LPS in BV2 cells (Yang et al., 2020; Hung et al., 2022) and primary rat microglia (Zhang et al., 2015), as well as the responsiveness to IFN-γ in human macrophages by boosting the STAT1 phosphorylation, the expression of metalloproteases1 and IL-1β secretion (Nareika et al., 2009), and in human endothelial cells resulting in the activation of p38MAPK/COX_2_/EP_1_/PLC/IP_3_ cascades, increased production of IL-1β, TNF-α, ATP and NO, cytosolic Ca^2+^ signaling, and the opening of Cx43 hemichannels (Dosch et al., 2019; Sáez et al., 2020). The IFN-γ-dependent inflammatory response was already proved on primary murine microglia, microglia in hippocampal slice cultures resulting in cognitive impairment and depression-like behavior, and on human iPSC-microglia to boost LPS-activation and recapitulate the LPS-dependent response described in mouse microglia (Lively and Schlichter, 2018; Ta et al., 2019; Zhang J. et al., 2020; Jiang et al., 2021; Kenkhuis et al., 2022; Monzón-Sandoval et al., 2022). Unlike the murine BV2 cells, in which the NLRP3/caspase/IL-1β pathway was found to be dependent on TREM2 upon high glucose stimulation, we found a low involvement of TREM2-dependent activation of Caspase1/IL-1β, confirming the less responsiveness of human cells to inflammatory stimuli, and the different sensitivity to glucose compared to murine cells, thus justifying the coupling glucose plus IFN-γ (Li Y. et al., 2021). On the one hand, TREM2 inhibits the NF-κB-dependent inflammatory response in mouse microglia (Alexandrov et al., 2013; Li et al., 2019), and in bone marrow-derived macrophages (Wang et al., 2022) as well as the Aβ induced apoptosis in human microglia (Akhter et al., 2021), on the other hand, the increased glycolytic rate was reported to be responsible for the increased transcription of NF-κB-dependent pro-inflammatory genes (Shen et al., 2017). Nevertheless, the combination of IFN-γ plus high glucose did not influence the NF-κB(p65) phosphorylation in our cells. This can be due either to the TREM2-dependent modulation of the inflammatory response or to the inability of HMC3 cells to produce the TNF-α, found to induce the NF-κB(p65) phosphorylation in macrophages (Sakurai et al., 2003). The involvement of TREM2 in the immunomodulation of HMC3, while maintaining the phagocytic properties (Zhang et al., 2018), together with the unchanged expression of Arginase 1, recapitulated the dynamic and continuous polarization of HMC3 cells, also reported upon exposure to chronic hyperglycemia, from the early M2a phenotype to the M2b one, up to the pro-inflammatory polarization, as function of ERK5 pathway (Chen et al., 2019). Additionally, the unchanged expression of Arginase 1 upon IFN-γ stimulation was also demonstrated in primary murine microglia in which IL-4 not IFN-γ was able to modulate its expression, as opposed to iNOS, together with the IBA response (Rui et al., 2020; Chauhan et al., 2021; Jiang et al., 2021), leading us to speculate about an early shift toward the pro-inflammatory phenotype in our model also confirmed by the morphological analysis of IBA1^+^ cell, indicating a less ramified, ameboid phenotype (Bachstetter et al., 2015; Davis et al., 2017; Jurga et al., 2020; Rai et al., 2020; Leyh et al., 2021). So, we demonstrated the pro-inflammatory polarization of HMC3, while preserving the phagocytic function, exploiting the priming effect of IFN-γ enhanced by the high glucose treatment.

After, we demonstrated the microglia polarization in response to Parkinsonian stimuli aiming at developing a human cell model able to recapitulate the neuroinflammation found in PD patients (Liu et al., 2022).

We showed a significant upregulation of iNOS expression and the following nitrite release in HMC3 cells upon treatment with 6-OHDA and MPTP, consistent with the pro-inflammatory activation found *in vitro* (Yan et al., 2015; Zhang et al., 2017) and *in vivo* (Dehmer et al., 2000, 2004; Yao and Zhao, 2018; Li M. et al., 2021), where the effect of the neurotoxins is associated with a reduced number of TH^+^ neurons restored by the modulation of the inflammatory response through iNOS and NF-κB silencing and overexpression of TREM2 (Dehmer et al., 2000; Gasparotto et al., 2017; Ren et al., 2018; Zhang et al., 2018). Nevertheless, we observed no changes in either the expression of TREM2 nor Arginase1, although their upregulation was found in MPTP–mice as a possible compensatory mechanism to counteract neuroinflammation through the TREM2/ULK1-autophagy-dependent pathway. However, knockdown of TREM2 in BV2 microglia inhibited the anti-inflammatory polarization leading to exaggeration of inflammatory responses (Zhang et al., 2018; Lv et al., 2021). Interestingly, the *in vivo* upregulation of TREM2 found few days after MPTP injection was decreases to control level in accordance with the reduction of TH^+^neurons and the anti/pro-inflammatory shift of microglia in the late stage of the pathology, as well as in MPTP-aged mice compared to MPTP-young ones as function of upregulated iNOS and downregulated arginase-1 expression, exacerbating the neurodegeneration (Ren et al., 2018; Yao and Zhao, 2018). This evidence validated the inflammatory profile of our Parkinson’s like HMC3 models, which partially retain phagocytic properties. We then confirmed that the MPTP-dependent pro-inflammatory phenotype was induced by the nuclear translocation of NF-κB as previously elucidated in rat astrocytoma cells and in a mouse model of MPTP-PD (Dehmer et al., 2004; Niranjan et al., 2010; Ghosh et al., 2018). Even though the NF-κB pathway seems to be a common mechanism engaged by dopaminergic neurotoxins, as demonstrated by the presence of NF-κB(p65) in the *substatia nigra* (SN) of 6-OHDA-rats, in our *in vitro* 6-OHDA-microglia we did not found a comparable involvement (Gasparotto et al., 2017; Machado et al., 2019). This might be attributable to an overestimation of microglial contribution in the quantification of nuclear p65 in rat SN and/or a different mechanism that established the neuroinflammation reaction triggered by MPTP or 6-OHDA. While the ERK1/2/MAPKs and NF-κB pathways, as well as the NLRP3 inflammasome have been found in MPTP-models, the prostaglandin mPGES-1/PGE2 and the Phosphodiesterase seven might contribute to the pathogenic mechanism initiated by 6-OHDA (Fathi et al., 2022). Although the NF-κB signaling might be regulated by Caspase1 (Staal et al., 2011), responsible for triggering a pyroptotic and/or a caspase-7/PARP1/AIF apoptotic death *in vivo*, we did not found any involvement of the protease in HMC3 cells, in line with our aim to induce an inflammatory phenotype rather than a toxic condition (Qiao et al., 2017; Rui et al., 2020; Zhang C. et al., 2021; Cai et al., 2022). Also, in most *in vitro* studies, NF-κB/Caspase1 pathway was usually studied in LPS-activated cells; only one recent study reported the upregulation of Caspase1 in primary mixed glial cells, induced by concentrations of MPTP toxic for our cells and otherwise linked to pyroptosis (Rui et al., 2020). Thus, we can speculate about a different mechanism of action between 6-OHDA and MPTP *in vitro* in human microglia. Additionally, unlike the abovementioned treatments, rotenone has not brought to any phenotypic change on our HMC3 cells, not even in combination with IFN-γ or glucose. It can be due to a different mechanism to impair microglial function, e.g., the autophagy-dependent JNK-pathway (Wu et al., 2015; Liang et al., 2020), a lower response of microglia to rotenone in comparison to MPTP, as found *in vivo* (Bhurtel et al., 2019), and *in vitro* (Gao et al., 2013; Liang et al., 2015; Zhang Q. et al., 2020). Finally, the increased IBA1 fluorescence intensity and the reduced cells size upon Parkinson’s like stimuli, confirmed the functional pro-inflammatory polarization of HMC3 cells, still retaining the ameboid morphology of myeloid cells already demonstrated by the preserved expression of TREM2 and Arginase1 (Bachstetter et al., 2015; Haas et al., 2016; Davis et al., 2017; Bhurtel et al., 2019; Chen et al., 2019; Jurga et al., 2020; Ye et al., 2020; Leyh et al., 2021; Zhang D. et al., 2021).

In conclusion, we established new models of Parkinson’s like neuroinflammation in human microglia in response to the mitochondrial inhibitors 6-OHDA and MPTP, as function of NF-κB in the latter, and the priming effect of IFN-γ plus boosted by high glucose treatment.

## Data availability statement

The raw data supporting the conclusions of this article will be made available by the authors, without undue reservation.

## Author contributions

FD performed the experiments and analyzed the data. EP, GB, and IP performed the experiments. BM conceptualized the approach and designed the experiments, revised, and edited the manuscript. FM conceptualized the approach and designed the experiments, analyzed, and interpreted the data, drafted, revised, and edited the manuscript. All authors contributed to the article and approved it for publication.
