# Supplementary material for: New models of Parkinson’s like neuroinflammation in human microglia clone 3: Activation profiles induced by INF-γ plus high glucose and mitochondrial inhibitors
Source: Front Cell Neurosci. 2022 Nov 29;16:1038721. doi: 10.3389/fncel.2022.1038721 (PMC9744797; doi:10.3389/fncel.2022.1038721)
Supplement: Supplementary file 1 [file Presentation_1.pdf]

Supplementary Material

1. Supplementary Figure

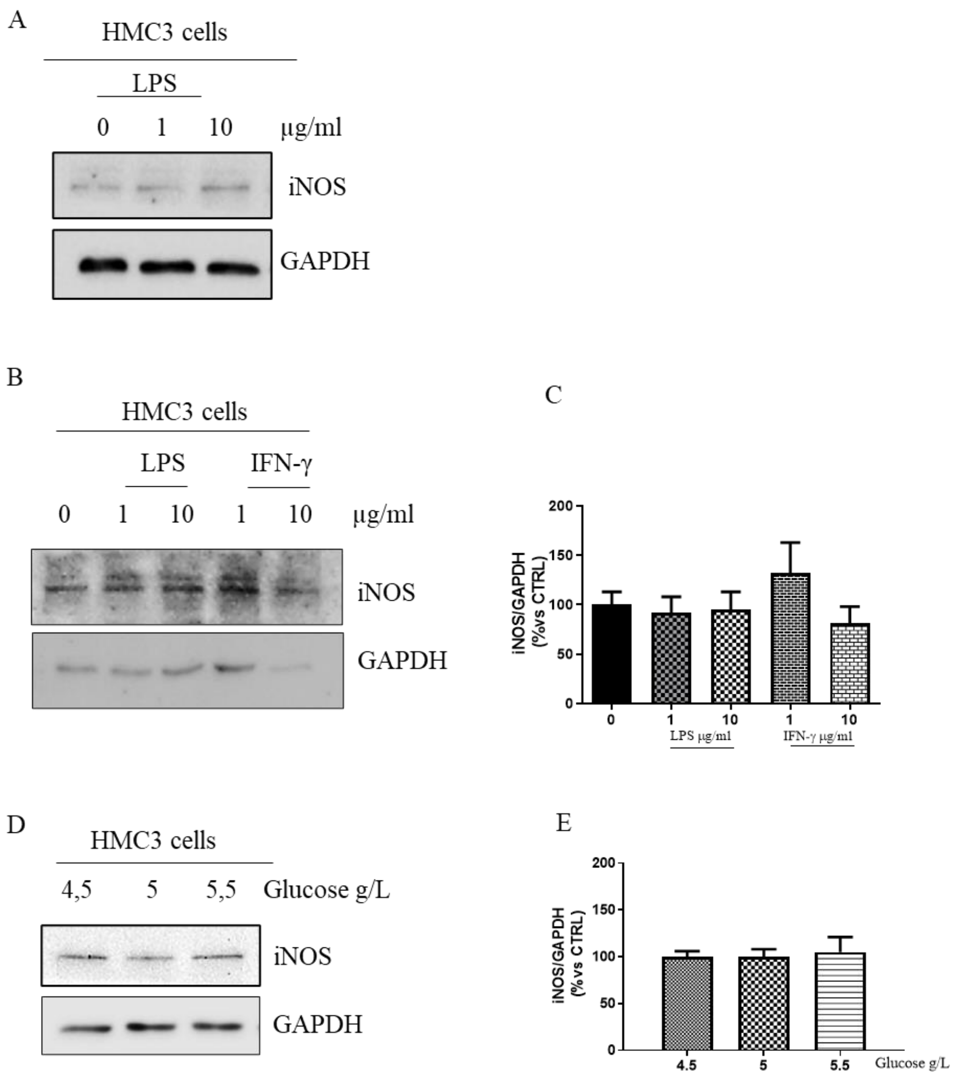

Supplementary Figure 1. Human microglial clone 3 cell line (HMC3) activation upon treatment with increasing concentration of LPS or IFN-γ or high glucose

Western blot analysis and relative densitometry of iNOS vs the reference protein GAPDH in HMC3 cells treated with increasing concentration of LPS or IFN-γ (A,B,C) (0,1,10 µg/ml) and increasing concentration of glucose (C,D) (4,5, 5, 5,5 g/l) for 24h in serum free medium (4,5 g/l glucose). N=3 ± s.e vs % CTRL.

2. Supplementary Figure

A

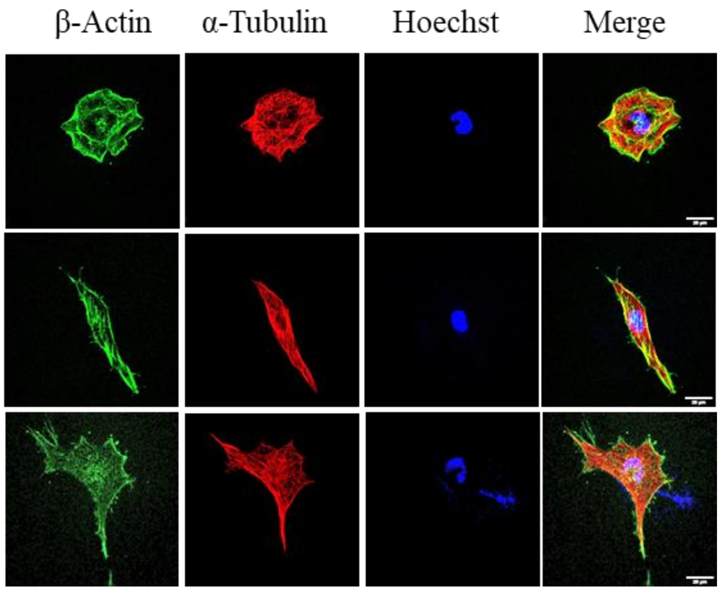

Supplementary Figure 2. Morphological characterization of human microglial clone 3 cell line

(A) Microglial cells stained for cytoskeletal proteins β-Actin (green) and α-Tubulin (red). Nuclei were stained with Hoechst (blu). 100X oil immersion objective. Scale bar=20μm.

3. Supplementary Figure

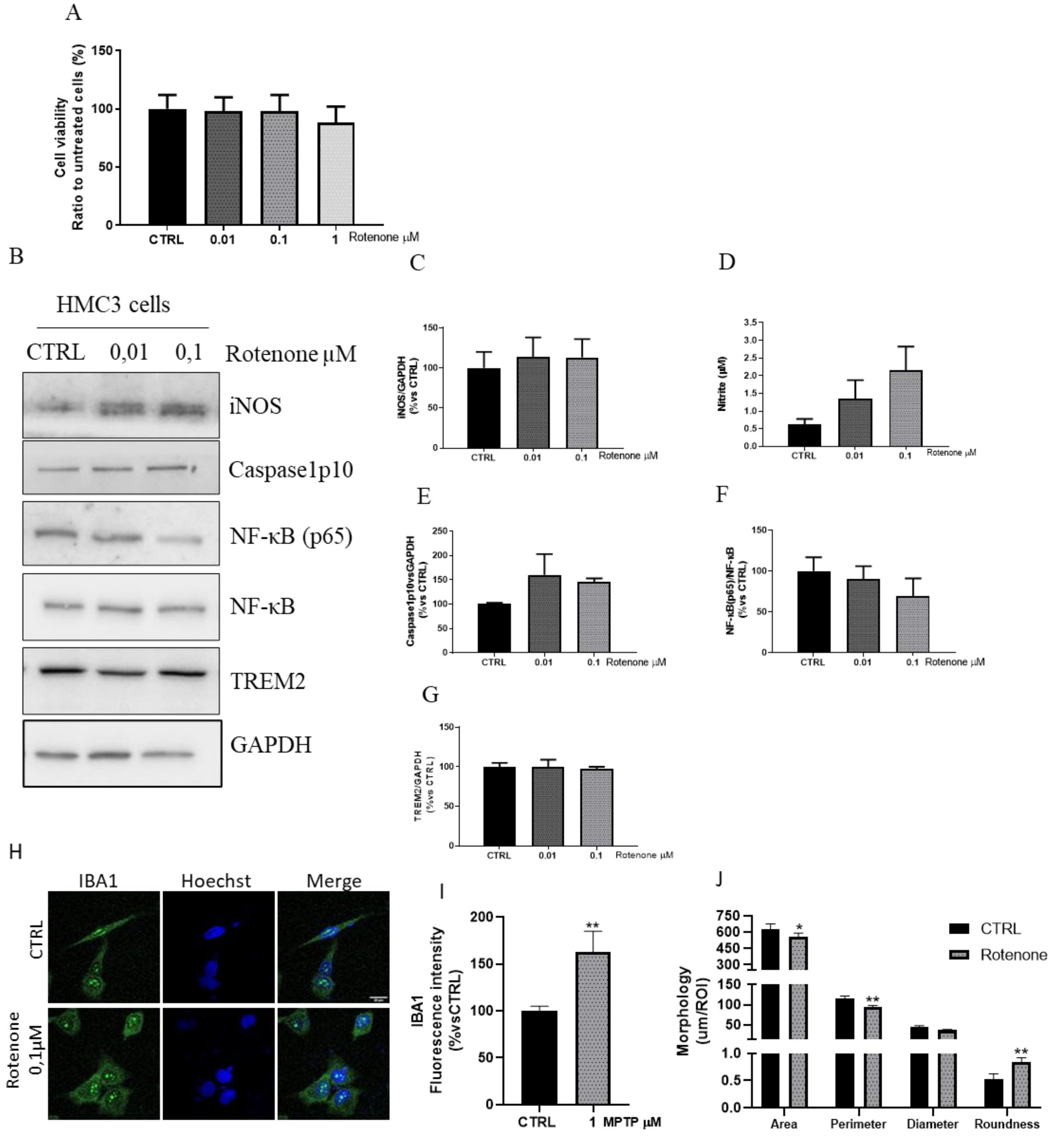

Supplementary Figure 3. Phenotypic markers expression in human microglial clone 3 cell line (HMC3) treated with increasing concentration of Rotenone

(A) Cell viability MTT assay for HMC3 cells treated with increasing concentration of Rotenone (0,01, 0,1, 1 µM) for 24h in serum free medium. Western blot analysis and relative densitometry of iNOS (B,C), Caspase1p10 (B,E), NF-κB(p65)/ NF-κB (B,F), TREM2 (B,G), in lysed cells vs the reference protein GAPDH and nitrite quantification in conditioned medium (D) of HMC3 cell treated with increasing concentration of Rotenone (0,01, 0,1 µM) for 24h in serum free medium. Representative immunofluorescence of IBA1 (H) and relative quantification of fluorescence intensity (I) and morphology analysis (J). Results are the mean of 2/3 independent experiments ± s.e. vs CTRL. One way ANOVA followed by Dunnett's test *p<0,05, **p<0,01 vs CTRL.

.

4. Supplementary Figure

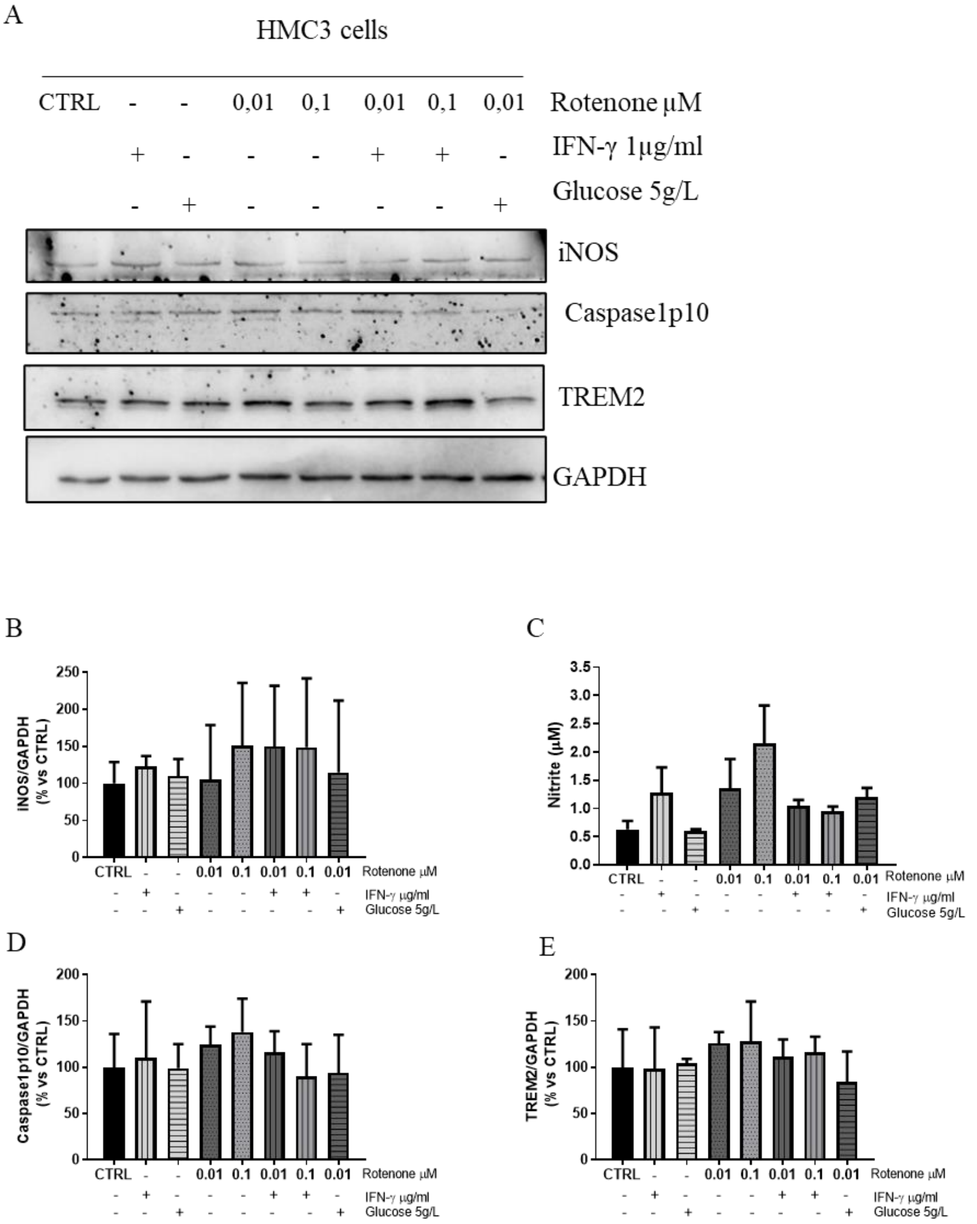

Supplementary Figure 4. Phenotypic markers expression in human microglial clone 3 cell line (HMC3) treated with increasing concentration of Rotenone w or w/o IFN-γ or glucose

(A) Western blot analysis and relative densitometry of iNOS (A,B), Caspase1p10 (A,C), TREM2 (A,E), in lysed cells vs the reference protein GAPDH and nitrite quantification in conditioned medium (C) of HMC3 cell treated with increasing concentration of of Rotenone (0, 0,01, 0,1 µM, w/o IFN-γ or glucose) for 24h in serum free medium. Results are the mean of 2 independent experiments ± s.e. vs CTRL.

5. Supplementary Figure

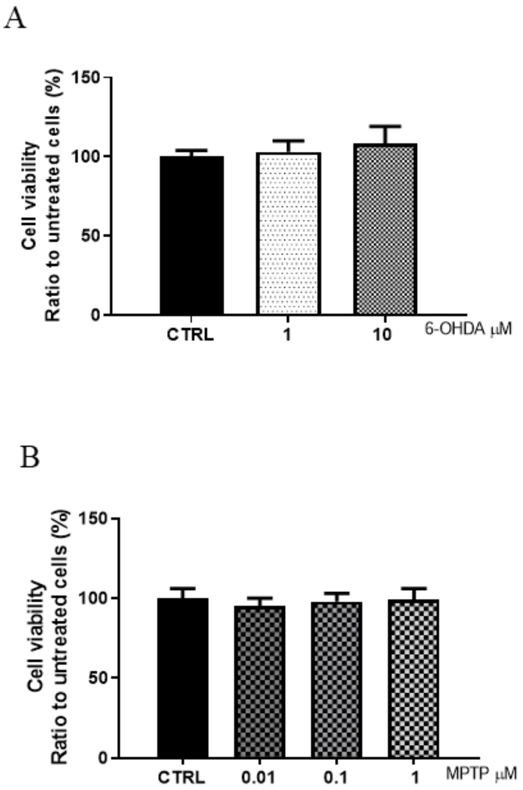

Supplementary Figure 5. Cell viability of human microglial clone 3 cell line (HMC3) treated with inflammatory mediators

Cell viability MTT assay for HMC3 cells treated with increasing concentration of 6-OHDA (0, 1, 10 µM) (A), and MPTP (0, 0,01, 0,1, 1 µM) (B) for 24h in serum free medium. Results are the mean ± s.e. of 3 independent experiments run in quadruplicate vs % CTRL
